# An Ancestral Retrovirus Envelope Protein Regulates Persistent Gammaherpesvirus Lifecycles

**DOI:** 10.3389/fmicb.2021.708404

**Published:** 2021-08-09

**Authors:** Tiffany R. Frey, Ibukun A. Akinyemi, Eric M. Burton, Sumita Bhaduri-McIntosh, Michael T. McIntosh

**Affiliations:** ^1^Department of Pediatrics, Child Health Research Institute, University of Florida, Gainesville, FL, United States; ^2^Division of Infectious Diseases, Department of Pediatrics, University of Florida, Gainesville, FL, United States; ^3^Department of Molecular Genetics and Microbiology, University of Florida, Gainesville, FL, United States

**Keywords:** Epstein-Barr virus, Kaposi’s sarcoma associated herpes virus, latency, lytic activation, epigenetics, endogenous retrovirus, Syncytin-1

## Abstract

Human gammaherpesviruses Epstein-Barr virus (EBV) and Kaposi’s sarcoma-associated herpesvirus (KSHV) persist as life-long infections alternating between latency and lytic replication. Human endogenous retroviruses (HERVs), via integration into the host genome, represent genetic remnants of ancient retroviral infections. Both show similar epigenetic silencing while dormant, but can reactivate in response to cell signaling cues or triggers that, for gammaherpesviruses, result in productive lytic replication. Given their co-existence with humans and shared epigenetic silencing, we asked if HERV expression might be linked to lytic activation of human gammaherpesviruses. We found *ERVW-1* mRNA, encoding the functional HERV-W envelope protein Syncytin-1, along with other repeat class elements, to be elevated upon lytic activation of EBV. Knockdown/knockout of *ERVW-1* reduced lytic activation of EBV and KSHV in response to various lytic cycle triggers. In this regard, reduced expression of immediate early proteins ZEBRA and RTA for EBV and KSHV, respectively, places Syncytin-1’s influence on lytic activation mechanistically upstream of the latent-to-lytic switch. Conversely, overexpression of Syncytin-1 enhanced lytic activation of EBV and KSHV in response to lytic triggers, though this was not sufficient to induce lytic activation in the absence of such triggers. Syncytin-1 is expressed in replicating B cell blasts and lymphoma-derived B cell lines where it appears to contribute to cell cycle progression. Together, human gammaherpesviruses and B cells appear to have adapted a dependency on Syncytin-1 that facilitates the ability of EBV and KSHV to activate lytic replication from latency, while promoting viral persistence during latency by contributing to B cell proliferation.

## Introduction

Human gammaherpesviruses Epstein-Barr virus (EBV; Human herpesvirus 4) and Kaposi’s sarcoma-associated herpesvirus (KSHV; Human herpesvirus 8) cause persistent, life-long infections (Knipe and Howley, 2013). This is due to their adaptation and sophisticated exploitation of human physiology and molecular pathways. Like other herpesviruses, EBV and KSHV have a biphasic life cycle consisting of a lytic or productive phase and a latent or quiescent phase (Knipe and Howley, 2013). Regulation of these phases is central to their immune evasion, transmission, and pathogenesis in acute and chronic diseases as well as in associated lymphoproliferative diseases (LPD), lymphomas, and epithelial cell cancers (Thorley-Lawson and Gross, 2004; Knipe and Howley, 2013; Thorley-Lawson, 2015; Aneja and Yuan, 2017; Broussard and Damania, 2020). Both viruses are known to persist latently in B lymphocytes with periodic lytic (re)activation producing virus particles that can infect new B cells, expanding the pool of latently infected B cells or epithelial cells, supporting transmission to new hosts (Hong et al., 2005; Kalla et al., 2010; Knipe and Howley, 2013; Broussard and Damania, 2020). This contributes to high prevalence rates of 1–60% for KSHV among distinct populations and a near ubiquitous presence of EBV with greater than 90% prevalence overall (Henke-Gendo and Schulz, 2004; Knipe and Howley, 2013).

While EBV and KSHV generally persist as asymptomatic infections in immunocompetent individuals, EBV can cause infectious mononucleosis, frequently upon primary infection of adolescents, and both are causal to a number of lymphomas and cancers (Knipe and Howley, 2013). EBV causes endemic African Burkitt lymphoma (BL), Hodgkin lymphoma, nasopharyngeal cell carcinoma, and some gastric cancers (Knipe and Howley, 2013). More frequently, in immunocompromised individuals and transplant recipients, EBV causes diffuse large B cell lymphoma and B cell LPD, as well as more rarely T and natural killer (NK) cell lymphomas (Knipe and Howley, 2013; Li and Bhaduri-McIntosh, 2016). Similarly, KSHV causes Kaposi’s sarcoma, an immunosuppression-associated multifocal cancer derived from vascular endothelial cells (Bhaduri-McIntosh, 2005; Knipe and Howley, 2013). KSHV combined with severe immunocompromise is also associated with primary effusion B cell lymphoma (PEL), HHV8-associated germinotropic LPD, and multicentric Castleman disease LPD and lymphoma (Bhaduri-McIntosh, 2005; Knipe and Howley, 2013; Li and Bhaduri-McIntosh, 2016).

Epstein-Barr virus and KSHV have linear dsDNA genomes, ca. 170 kb and 140 kb in size, respectively, with nearly 100 genes each (Knipe and Howley, 2013). Most of the genes encode proteins or RNA that function during lytic production of virus. During latency, however, the multicopy nuclear localized viral genomes circularize as episomes with a mere few protein-coding genes, and/or miRNA and lncRNA maintaining expression to promote cell survival, proliferation, and viral latency (Knipe and Howley, 2013; Kempkes and Robertson, 2015; Broussard and Damania, 2020). This silencing is critical to immune evasion and viral persistence.

Epigenetic modifications that silence EBV and KSHV genomes in latency include histone methylation, histone deacetylation, and CpG methylation that promote heterochromatinization and transcriptional silencing (Scott, 2017; Pei et al., 2020; Bhaduri-McIntosh and McIntosh, 2021). A key regulator is the ubiquitous cellular transcriptional co-repressor Krüppel-associated Box-associated protein 1 or tripartite motif-containing protein 28 (KAP1/TRIM28) (Groner et al., 2010; Cheng et al., 2014; Jang et al., 2018). KAP1 is recruited to DNA via binding to selective members of a large family of KRAB domain-containing zinc finger proteins that exhibit sequence/locus specific DNA binding (Cheng et al., 2014; Bhaduri-McIntosh and McIntosh, 2021). KAP1 then recruits histone modifying enzymes and DNA methyl transferases to epigenetically silence select regions of the genome (Groner et al., 2010; Cheng et al., 2014; Jang et al., 2018; Bhaduri-McIntosh and McIntosh, 2021). Previously, we and others have demonstrated that KAP1 also silences the genomes of EBV and KSHV, as well as the betaherpesvirus cytomegalovirus (CMV) during latency (King et al., 2015; Rauwel et al., 2015; Li et al., 2017, 2018, 2019b; Burton et al., 2020, 2021; Bhaduri-McIntosh and McIntosh, 2021). In response to lytic cycle triggers/cues, suppression of cellular KAP1 by depletion triggers the lytic switch protein ZEBRA or via phosphorylation enhances expression of ZEBRA, leading to activation of lytic replication from latency (King et al., 2015; Li et al., 2017, 2019b; Burton et al., 2020, 2021; Bhaduri-McIntosh and McIntosh, 2021). *Bam*HI Z EBV replication activator (ZEBRA) and replication and transcription activator (RTA) in EBV, or RTA in KSHV, are immediate early viral transcription factors, also known as latent-to-lytic switch proteins, that initiate transcription of the vast majority of viral genes in a cascade that proceeds kinetically with early lytic genes (pre viral DNA replication) to late lytic genes (post viral DNA replication) (Kenney and Mertz, 2014; Broussard and Damania, 2020).

Retroviruses likewise cause persistent infections though facilitated by frequent integration of proviral cDNA into the genome of the infected cell (Knipe and Howley, 2013). Over the course of our evolution, some have become fixed within the human germline, known as human endogenous retroviruses (HERVs) (Nelson et al., 2003; Johnson, 2019). Their ability to retrotranscribe and reinsert has resulted in formation of repetitive DNA elements that account for more than 8% of the human genome (Lander et al., 2001). In addition, many of these replication-competent elements re-infected the germline throughout evolution, resulting in multiple copies of some HERVs (Belshaw et al., 2004, 2005; Marchi et al., 2014). However, at present, we know little about their impact on our biology (Nelson et al., 2003; Johnson, 2019). Importantly, HERVs are also silenced by KAP1-mediated heterochromatinization (Rowe et al., 2013; Hurst and Magiorkinis, 2017; Yang et al., 2017; Wolf et al., 2020) and aberrant expression of HERVs, and in particular *ERVW-1*, encoding the functional relic of a viral envelope protein, Syncytin-1, are associated with a variety of cancers and autoimmune/inflammatory conditions (Bjerregaard et al., 2006; Maliniemi et al., 2013; Grandi and Tramontano, 2018; Wang et al., 2018; Gao et al., 2021). Syncytin-1 also has an exapted role in mediating cell-to-cell fusion of placental trophoblasts during development, essential to human reproduction (Johnson, 2019).

Given the co-existence of HERVs, gammaherpesviruses and humans, and shared KAP1-mediated epigenetic silencing, we asked if the expression of HERV elements might be linked to lytic activation of gammaherpesviruses. We found that low-level transcription of *ERVW-1* in particular was consistently elevated during lytic activation of EBV. This led us to look more closely at the potential influence of Syncytin-1 on gammaherpesvirus infections in various lymphoma and lymphoblastoid B cell lines. Surprisingly, our results find Syncytin-1 expressed at a basal level in B lymphoma cells and replicating B cell blasts. Cells supporting lytic replication of EBV or KSHV derive from B cells with a higher expression level of Syncytin-1, and modulating Syncytin-1 levels in B cells further regulates the ability of EBV and KSHV to activate lytic replication from latency.

## Materials and Methods

### Cell Lines and Chemical Treatment

EBV^+^ Burkitt lymphoma cell lines Akata (a gift from Ben Gewurz, Harvard University), HH514-16 (a gift from Dr. George Miller, Yale University), lymphoblastoid cell lines (LCL) created as described previously (Hui-Yuen et al., 2011), and KSHV^+^ primary effusion lymphoma cell line BCBL-1 (a gift from Dr. Christine King, SUNY Upstate Medical University) were cultured in RPMI 1640 (Gibco) supplemented with 10% fetal bovine serum (Alphabioregen) and 1% penicillin-streptomycin (Gibco). Akata cells were maintained in Geneticin (700 μg/mL; 11811023; Gibco) as a selection marker to maintain the EBV genome. HH514-16 and LCL were treated with sodium butyrate (NaB, 3 mM; 303410, Sigma-Aldrich) or 5-AZA-2′-deoxycytidine (Aza, 5 μM; A3656, Sigma-Aldrich), and Akata cells were treated with rabbit anti-human IgG (1:200 dilution; A042301-2, Dako) for lytic induction studies. For lytic induction of KSHV, BCBL-1 cells were treated with 12-O-Tetradecanoylphorbol 13-acetate (TPA; 32 mM; P8139, Sigma-Aldrich) or valproic acid (VPA; 0.6 μM, P4543, Sigma-Aldrich). CLIX-sh*ERVW1* (clone HH514-16 transfected with pLIX_sh*ERVW1*) cells were generated from puromycin (P8833; Sigma-Aldrich)-selected HH514-16 cells transfected with pLIX_402-sh*ERVW1*. These cells were treated with 10 μg of doxycycline to induce *ERVW-1* knockdown (D9891; Sigma-Aldrich).

### Antibodies

Antibodies used in this study include rabbit anti-Syncytin-1 (for western blot, PA522819; Invitrogen), rabbit anti-Syncytin-1 (for flow cytometry, PA522819; Invitrogen), mouse anti-bZIP (sc69797; Santa Cruz), mouse anti-K8.1 (sc65446; Santa Cruz), rabbit anti-KSHV RTA (a gift from Yoshihiro Izumiya at University of California Davis), mouse anti-β-actin (AC-15; Sigma), rabbit anti-GAPDH (2118S; Cell Signaling), mouse anti-EA-D (MAB8186; EMD), mouse IgG1 negative isotype (CBL610; EMD Millipore), mouse anti-ZEBRA (a gift from Paul Farrell at Imperial College London, London, United Kingdom), rabbit anti-FLAG (F7425; Millipore), rat anti-EBNA2 (MABE8; Millipore), mouse anti-BrdU (555627; Thermo Fisher Scientific), mouse anti-CD19-APC (MABF197; Sigma-Aldrich), mouse anti-CD20-PE (MABF1635; Sigma-Aldrich), mouse IgG1-APC isotype (550824; BD Pharmingen), rat IgG2a isotype (MABF1077Z; EMD Millipore), horseradish peroxidase (HRP)-conjugated goat anti-mouse IgG (H + L) (AP308P; EMD Millipore), HRP-conjugated goat anti-rabbit IgG (AP307P; EMD Millipore), FITC-conjugated goat anti-mouse IgG (F0257; Sigma), and APC-conjugated goat anti-rabbit IgG. All antibodies were used at concentrations and conditions recommended by manufacturers.

### Nascent Transcriptome Analysis

Nascent transcription was previously determined using Bru-Seq at 3, 24, and 48 h post exposure of EBV^+^ HH514-16 cells to the lytic inducing agent, sodium butyrate (NaB), as described (Frey et al., 2020). Raw Bru-Seq reads were downloaded from the NCBI’s Gene Expression Omnibus under accession number GSE141220. Low quality reads and adapter sequences were removed and trimmed with Trimmomatic version 0.39 (Bolger et al., 2014). Clean reads were mapped to the repeat masked human genome, Genome Reference Consortium Human Build 38 (GRCh38) using Bowtie 2 (Langmead and Salzberg, 2012). Unaligned reads were then analyzed with RepeatMasker version 4.1.1 (Smit, AFA, Hubley, R. & Green, P “RepeatMasker” at http://www.repeatmasker.org), and repeat elements were annotated using the Dfam database version 3.0 (Storer et al., 2021). Fold change was calculated as the simple ratio of induced to uninduced reads. Induced reads were normalized to total reads in uninduced control samples, and data represent the means of two independent experiments.

### siRNAs, Plasmids, and Transfection

siRNAs targeting *ERVW-1* were custom designed and purchased from Qiagen and reconstituted with nuclease free water at a concentration of 10 μM. The sense sequences for the siRNAs were: scrambled control siRNA: UUCUCCGAAC GUGUCACGU; *ERVW-1* siRNA 1: UAGGUGCACUAGGUAC UGGTT; *ERVW-1* siRNA 2: UCGAAGAGCUUUAGACUUG. *ERVW-1* CRISPR all-in-one vector was purchased from Genscript. All-in-one vector was created in the pSpCas9 BB-2A-Puro backbone to target the following sequence of the coding region: CCCCATCAGACATACCAGTT. pcDNA-Syncytin-1 FLAG was codon optimized and purchased from Addgene. One million Akata, HH514-16, or BCBL-1 cells were transfected with 200 pmoles of siRNA or 20 μg of plasmid DNA in 100 μL Ingenio solution (MIR50117; Mirus) using an Amaxa Nucleofector II (Program A-024) as described followed by seeding at 5 × 10^5^ in pre-warmed RPMI 1640. siRNA experiments were performed using two unique siRNA sequences with representative data shown.

### Primer Design and Quantitative PCR (qPCR)

Primers sequences for the analysis of *ERVW-1* transcription are as follows: Forward (5′ to 3′): TGCCCCATCGTATAGGAGTCT, Reverse (5′ to 3′): CATGTACCCGGGTGAGTTGG. Primers used for analysis of EBV genes have been previously described (Hill et al., 2013). Reverse transcriptase-quantitative PCRs were performed and analyzed using the ΔΔCT method as described (Schmittgen and Livak, 2008). All data were normalized to the *18S* endogenous cellular control.

### Immunoblotting

Akata, HH514-16, LCL, or BCBL-1 cells were harvested after the respective time points, lysed, and immunoblots were performed as previously described (Koganti et al., 2014) using primary antibodies of interest. Mouse anti-β-actin (AC-15; Sigma) or rabbit anti-GAPDH (2118S; Cell Signaling) were used as cellular loading controls. All immunoblotting was performed using 10% SDS-PAGE gels. Densitometry values were calculated by comparing protein of interest to β-actin or GAPDH controls of the same sample before normalizing to the control condition using Image Studio software.

### Flow Cytometry

HH514-16 cells were flow cytometrically sorted into lytic and latent populations as previously described (Bhaduri-McIntosh and Miller, 2006; Hill et al., 2013). For all other flow cytometric analyses, cells were fixed with BD Cytofix/Cytoperm solution (554722; BD Bioscience) at room temperature for 20 min, washed twice with 1X BD Perm/Wash buffer (554723; BD Bioscience) and incubated with primary antibody for 45 min at room temperature. Cells were washed twice prior to incubation with a secondary antibody conjugated to a fluorophore for another 45 min at room temperature. Flow cytometry was subsequently performed using an Attune NxT Flow cytometer (Invitrogen). Antibodies were used at concentrations suggested by manufacturers. Data were analyzed using FlowJo software (Tree Star). Gates for flow cytometry were determined based on profiles of respective isotype-matched control-stained cells.

### Assay for EBV Load

Viral genomic DNA was prepared by extracting total DNA from HH514-16 cells using a DNeasy Kit (Qiagen). Released viral genomes were prepared by collecting supernatant from cell cultures followed by DNase treatment. EBV *BamW* gene was amplified via qPCR using the following primers: Forward (5′ to 3′): AGGCTTAGTATACATGCTTCTTGCTTT and Reverse (5′ to 3′): CCCTGGCTGATGCAACTTG. EBV genome copy numbers were calculated using a standard curve qPCR with BACmid p2089 serving as a template.

### Study Subjects and PBMC Infection

Peripheral blood mononuclear cells (PBMC) were isolated from the blood of two patients aged 6 and 14 years with elevated circulating EBV loads following kidney transplantation. Representative data are shown. PBMC were also isolated from three healthy subjects approximately 20 years of age. PBMC from healthy subjects were exposed to CD40L (50 ng/ml) and IL-4 (20 ng/ml) to generate EBV-negative B cell blasts or infected with EBV (B95-8 strain; MOI of 1–5) in the presence of 20 nM FK506, as previously described (Hui-Yuen et al., 2011). Cells were maintained in RPMI-1640 containing 10% fetal bovine serum and 1% penicillin/streptomycin and harvested at indicated time-points.

### Cell Cycle Analysis

For analysis of cell cycle, cells were harvested 3 h after incubation with 100 μM BrdU and stained as described for immunofluorescence. After incubation with primary and secondary antibodies, cells were washed and re-suspended in 150 μl of PBS containing 10 μg/ml RNase A (EN0531; Thermo Fisher Scientific) and 20 μg/ml propidium iodide (PI, P4864; Sigma) for 30 min at RT in the dark. Samples were then acquired using an Attune NxT Acoustic Focusing Cytometer (Invitrogen) and data were analyzed using FlowJo V10 software (Tree Star).

### Statistical Analysis

All *p-*values were calculated using an unpaired Student’s *t*-test via GraphPad Prism.

## Results

### Cells Expressing Higher Levels of Syncytin-1 More Readily Reactivate EBV

Using an EBV^+^ Burkitt lymphoma (BL) cell line in which EBV is tightly latent but can be induced into the lytic phase, we previously performed a nascent transcriptomic study to evaluate the regulation of host and EBV genomes (Frey et al., 2020). Induction of the lytic phase of EBV was accomplished by exposure of cells to the histone deacetylase inhibitor (HDACi) sodium butyrate (NaB) for 3, 24, or 48 h, prior to incorporation of 5-Bromouridine into nascent RNA followed by immune-capture and sequencing of nascent transcripts (Bru-Seq) (Frey et al., 2020). Analysis of Bru-Seq data against RepeatMasker and Dfam databases revealed differential regulation of several repeats including HERV/LTR elements (Supplementary Table 1). With the understanding that interpretation can be skewed between single and multi-copy loci depending on sequence similarity, pericentromeric satellite repeats displayed the most dramatic activation of transcription at 3, 24, and 48 h post lytic induction, while several HERV and LTR elements also appeared upregulated (Supplementary Table 1). HERV elements *ERVK13-1* and *ERV3-1* were among the most abundantly transcribed regardless of lytic induction while *ERVW-1* was among the most consistently upregulated HERV protein-coding elements at 24 h and 48 h post lytic induction (Supplementary Table 1).

**FIGURE 1:**
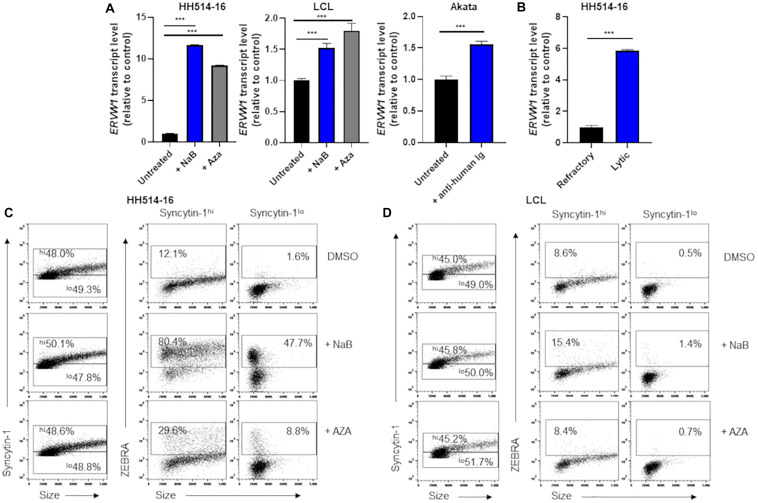
Cells high in Syncytin-1 more readily support the EBV lytic phase. **(A)** HH514-16, LCL, or Akata cells were treated with lytic inducing agents for 24 h and assayed for *ERVW-1* transcript levels using RT-qPCR. **(B)** HH514-16 cells were induced for 24 h with NaB, followed by FACS sorting to separate lytic from refractory cell populations and assayed for *ERVW-1* transcript via RT-qPCR. **(C,D)** Cells were induced as in **(A)** followed by flow cytometry staining for Syncytin-1 and ZEBRA protein. Syncytin-1 positive cells were sub-gated roughly equally into Syncytin-1^*hi*^ and Syncytin-1^*lo*^ sub-populations, which were then further examined for expression of ZEBRA. Data represent averages of three independent experiments; error bars, SEM; ***p ≤ 0.001.

With *ERVW-1* encoding a functional ancestral viral envelope glycoprotein and aberrant expression of this glycoprotein, Syncytin-1, associated with a number of malignancies and autoimmune conditions (Strick et al., 2007; Larsen et al., 2009; Sun et al., 2010; Mameli et al., 2012; Strissel et al., 2012; Mameli et al., 2013; Li et al., 2019a), we chose to examine it more closely in EBV^+^ BL cell lines HH514-16 and Akata, as well as in an EBV-derived B lymphoblastoid cell line (LCL). Within 24 h of exposure to either NaB or the DNA methyltransferase inhibitor 5-aza-2′-deoxycytidine (AZA), known triggers of EBV lytic replication, we observed increases in steady state levels of RNA from *ERVW-1* (Figure 1A). Exposure of the EBV^+^ BL cell line Akata for 24 h to anti-human immunoglobulin (IgG), which activates the lytic phase through B cell receptor crosslinking, likewise resulted in a statistically significant, though more modest, increase in *ERVW-1* transcription (Figure 1A). As many cells remain refractory to induction of the lytic cycle, and the effectiveness of lytic induction varies among host cell types and lytic triggers, we further assayed steady state levels of *ERVW-1* transcripts by RT-qPCR of sorted lytic and latently infected HH514-16 cells. This revealed a greater than 5 fold increase in steady state transcripts of *ERVW-1* in cells supporting lytic EBV replication (Figure 1B).

To assess Syncytin-1 expression on a cell-by-cell basis, we performed flow cytometry (Figures 1C,D). HH514-16 or LCL were exposed to lytic triggers NaB or AZA for 24 h followed by staining with antibodies targeting Syncytin-1 and ZEBRA (an immediate early EBV transcription factor that initiates the lytic phase). After gating on cells that were Syncytin-1 positive (virtually all live cells), we subdivided them into Syncytin-1 high and low populations and analyzed each for expression of ZEBRA to discriminate between cells that had initiated the lytic phase of EBV (ZEBRA positive) and cells that remained latently infected (ZEBRA negative) (Figures 1C,D). This revealed that cells expressing Syncytin-1 at high levels more readily give rise to cells harboring lytically replicating EBV (Figures 1C,D). This was evident even in cells not exposed to a lytic trigger (DMSO-treated) but still undergoing lytic EBV replication, referred to as spontaneous lytic (Figures 1C,D), suggesting that Syncytin-1 may serve as a pro-lytic factor in EBV infected B cells.

### Syncytin-1 Augments the EBV Lytic Cascade

**FIGURE 2:**
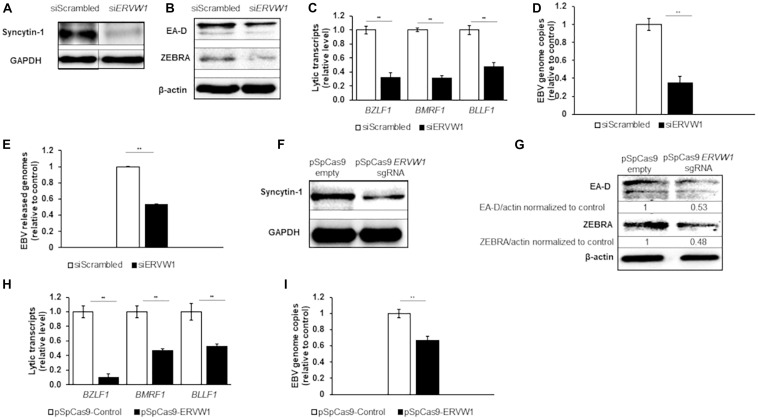
Depletion of Syncytin-1 impairs EBV lytic activation. Akata cells were nucleofected with scrambled control or *ERVW-1* specific siRNAs for 18 h and harvested for western blot analysis for Syncytin-1 **(A)** or treated with rabbit anti-human IgG to induce lytic activation and harvested at 24 h **(B)**, 36 h **(C,D)**, or 72 h **(E)** to analyze lytic readouts in each siRNA treatment group. Lytic proteins EA-D and ZEBRA were probed using western blot in B. Lytic transcript levels of representative genes of each EBV kinetic class were assayed using RT-qPCR in **(C)**. **(D)** EBV *BamW* qPCR was performed on DNA isolated from cell pellets. **(E)** EBV *BamW* qPCR was performed on DNase-treated, filtered cell culture medium. **(F–I)** Akata cells were nucleofected with pSpCas9 BB-2A-puro or pSpCas9 BB-2A-puro *ERVW-1* guide RNA for 24 h prior to harvest for western blot analysis **(F)** or treated with rabbit anti-human IgG to induce lytic activation. Cells were harvested at 24 h **(G)** or 36 h **(H,I)** to analyze lytic readouts as in **(B–D)**, respectively. Data represent averages of three independent experiments; error bars, SEM; **p ≤ 0.01.

The association between Syncytin-1 and EBV lytic activation was further examined by knockdown experiments. Using siRNA specific to *ERVW-1*, Syncytin-1 was depleted in Akata cells for 18 h followed by western blot analysis for confirmation (Figure 2A). At this time point, cells were treated with rabbit-anti human IgG to induce the lytic phase of EBV for 24, 36, or 72 h to assess effects of Syncytin-1 knockdown on viral lytic transcripts, lytic proteins, genome replication and virus release (Figures 2B–E). EBV lytic proteins ZEBRA and early antigen-diffuse (EA-D, viral DNA polymerase processivity factor) were noticeably reduced when Syncytin-1 was depleted (Figure 2B). Transcript levels for EBV genes of each kinetic class, including immediate early (*BZLF1*, encoding ZEBRA), early (*BMRF1*, encoding EA-D), and late (*BLLF1*, encoding viral envelope glycoprotein gp350), were likewise significantly reduced following Syncytin-1 knockdown (Figure 2C). Viral DNA replication and mature virus release were also assayed by qPCR of the EBV genome in cell lysates or filtered and DNase-treated culture media, respectively, revealing similar reductions in Syncytin-1 depleted cells (Figures 2D,E). These experiments, repeated in HH514-16 cells, yielded similar results (Supplementary Figure 1).

In a complementary approach to confirm the results obtained with siRNA, we utilized an all-in-one CRISPR plasmid system to knockout *ERVW-1*. Syncytin-1 was knocked down in Akata cells for 24 h by transient transfection of a CRISPR plasmid with a guide RNA to *ERVW-1*, followed by western blot analysis (Figure 2F). Cells were then treated with rabbit-anti human IgG as above. Similar to the siRNA experiments, EBV lytic proteins ZEBRA and EA-D were reduced (Figure 2G) as were EBV lytic transcripts from all three kinetic classes (Figure 2H) when Syncytin-1 levels were decreased. Viral DNA replication was also impaired following Syncytin-1 depletion (Figure 2I). When repeated in HH514-16 cells, these experiments again yielded similar results (Supplementary Figures 1E–I), thus revealing a functional dependency on Syncytin-1 for supporting or driving the lytic cycle of EBV.

**FIGURE 3:**
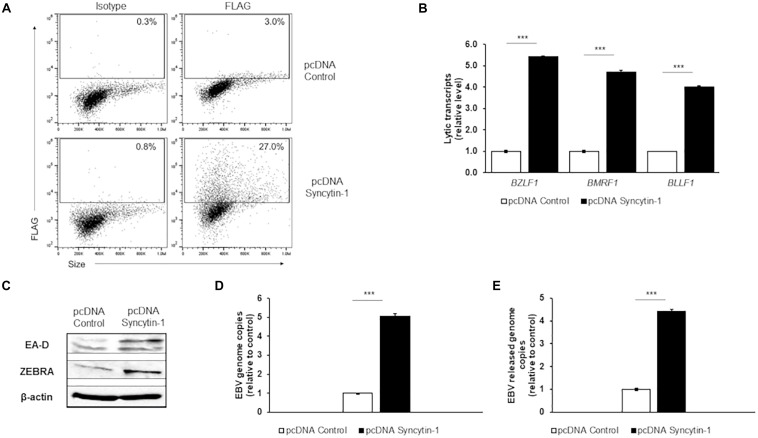
Syncytin-1 augments the EBV lytic cascade. Akata cells were nucleofected with empty vector pcDNA or FLAG-tagged pcDNA-Syncytin-1 and harvested 24 h later for flow cytometry to demonstrate Syncytin-1 overexpression via FLAG staining **(A)** or exposed to rabbit anti-human IgG to induce the lytic cycle and harvested after another 24 h **(C)**, 36 h **(B,D)**, or 72 h **(E)** to assay lytic activation by RT-qPCR of a representative lytic gene of each kinetic class **(B)**, immunoblotting with antibodies to indicated lytic antigens **(C)**, cell-associated viral load **(D)**, and DNase-resistant released virions **(E)**. Data represent averages of three independent experiments; error bars, SEM; ***p ≤ 0.001.

To explore Syncytin-1’s effect on the EBV lytic cascade further, we overexpressed Syncytin-1 with a C-terminal FLAG tag in EBV^+^ Akata BL cells. Following overexpression of Syncytin-1-FLAG for 24 hr, confirmed by flow cytometry (Figure 3A), EBV was induced into the lytic phase. RT-qPCR analysis of lytic genes again showed a significant increase in steady state viral mRNA this time in the Syncytin-1 overexpression condition compared to the empty vector control (Figure 3B). EA-D and ZEBRA protein expression were also enhanced (Figure 3C). Similarly, EBV genome copies increased approximately five fold, and DNase-resistant virus particles released into the culture medium increased ∼ 4.5 fold (Figures 3D,E). HH514-16 cell experiments using the same overexpression vector and lytic readouts with two different lytic cycle inducing agents (NaB and AZA) showed similar enhancement of lytic activation with Syncytin-1 overexpression (Supplementary Figure 2). Of note, overexpression of Syncytin-1 in the absence of a lytic trigger did not increase the number of spontaneous lytic cells (Supplementary Figure 3). Thus, taken together with the knockdown studies, these results reveal that Syncytin-1, while not sufficient as a direct trigger, indeed appears necessary for and promotes the lytic phase of EBV.

### KSHV Lytic Activation Is Also Dependent on Syncytin-1

**FIGURE 4:**
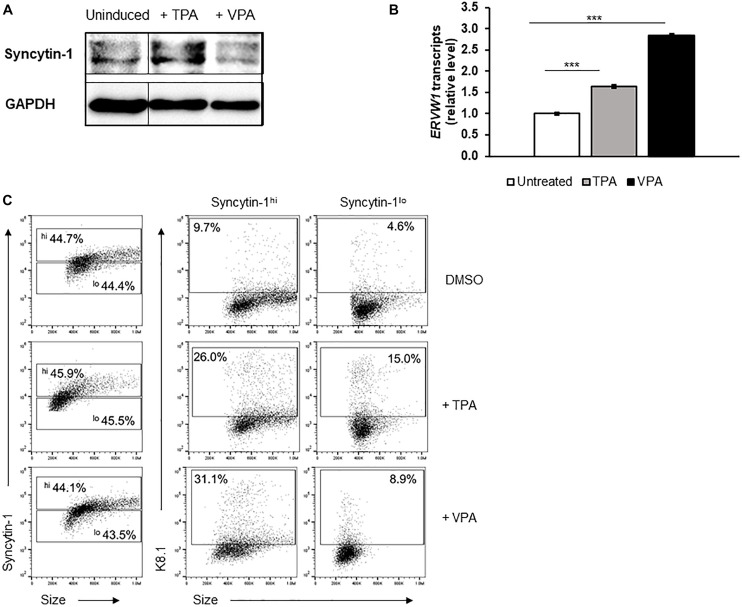
Cells high in Syncytin-1 more readily support the KSHV lytic phase. The KSHV^+^ PEL cell line BCBL-1 was exposed to TPA or VPA and harvested after 24 h **(A,B)** or 48 h **(C)** for immunoblotting **(A)**, RT-qPCR **(B)**, or flow cytometry **(C)**. In **(C)**, Syncytin-1 positive cells were sub-gated roughly equally into Syncytin-1^*hi*^ and Syncytin-1^*lo*^ sub-populations, which were then further examined for expression of the KSHV lytic K8.1 protein. Data represent averages of three independent experiments; error bars, SEM; ***p ≤ 0.001.

We next asked if the dependency on Syncytin-1 during EBV lytic replication could be conserved among human gammaherpesviruses. To address this, we investigated KSHV, a related cancer-causing gammaherpesvirus with a similar tropism for B lymphocytes. Using the KSHV + primary effusion lymphoma cell line BCBL-1 and inducing agents 12-O-tetradecanoylphorbol-13-acetate (TPA: a phorbol ester) or valproic acid (VPA: another HDACi), we again examined Syncytin-1 expression by western blot following 24 h treatments (Figure 4A). While clear elevation of total Syncytin-1 beyond a basal level was difficult to observe by western blot (Figure 4A), *ERVW-1* transcript levels were again significantly increased upon induction of lytic replication (Figure 4B). Flow cytometric analysis of uninduced, TPA-induced, or VPA-induced BCBL-1 cells again revealed that cells expressing high levels of Syncytin-1 give rise to a greater percentage of cells supporting lytic replication of KSHV regardless of the lytic trigger (Figure 4C), confirming that Syncytin-1 may serve as a pro-lytic factor for KSHV as well.

**FIGURE 5:**
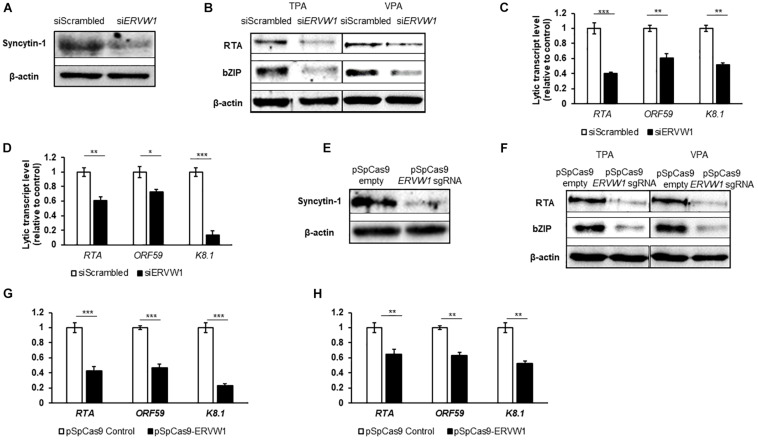
Syncytin-1 supports the KSHV lytic cascade. BCBL-1 cells were nucleofected with scrambled control or *ERVW-1* specific siRNAs for 18 h and harvested for western blot analysis for Syncytin-1 **(A)** or treated with TPA or VPA to induce lytic activation and harvested at 24 h **(B)** or 36 h **(C,D)** to analyze lytic readouts in each siRNA treatment group. Lytic proteins RTA and bZIP were probed using western blot in **(B)**. Lytic transcript levels of representative genes of each KSHV kinetic class were assayed using RT-qPCR after induction with TPA (C) or VPA **(D)**. **(E–H)** BCBL-1 cells were nucleofected with pSpCas9 BB-2A-puro or pSpCas9 BB-2A-*puro ERVW-1* guide RNA for 24 h prior to harvest for western blot analysis **(E)** or treated with TPA or VPA to induce lytic activation. Cells were harvested at 24 h **(F)** or 36 h **(G,H)** to analyze lytic readouts as in **(B–D)**, respectively. Data represent averages of three independent experiments; error bars, SEM; *p ≤ 0.05, **p ≤ 0.01, ***p ≤ 0.001.

Upon Syncytin-1 knockdown in BCBL-1 cells, confirmed by western blot (Figure 5A), cells were treated with either TPA or VPA for 24 or 48 h to determine if KSHV lytic replication displays a similar dependency on Syncytin-1. RT-qPCR analysis of one representative lytic gene from each KSHV kinetic class including RTA (encoding the replication and transcription activator), ORF59 (encoding a DNA polymerase processivity factor), and K8.1 (encoding a late lytic structural glycoprotein), showed a significant decrease in expression when Syncytin-1 was knocked down (Figures 5C,D). In addition, we assessed protein levels of two KSHV lytic proteins, RTA (the main latent-to-lytic switch regulator) and bZIP (a basic domain leucine zipper protein and regulator of RTA), encoded by immediate early and early genes, respectively (Figure 5B). Knockout using CRISPR-Cas9 targeting of *ERVW-1* was also performed. After confirmation of knockdown via western blot (Figure 5E), cells were treated with TPA or VPA for 24 or 48 h to assay for KSHV lytic genes and proteins. RT-qPCR analysis of RTA, ORF59, and K8.1 resulted in a decrease in lytic gene expression upon Syncytin-1 knockdown (Figures 5G,H). Protein levels of RTA and bZIP were also reduced (Figure 5F), confirming the phenotype seen with siRNA knockdown of Syncytin-1 (Figure 5B). As with EBV, Syncytin-1 knockdown reduced KSHV lytic protein expression in response to both inducing agents, supporting a shared role for Syncytin-1 in promoting lytic activation of both gammaherpesviruses.

### Syncytin-1 Also Augments the KSHV Lytic Cascade

**FIGURE 6:**
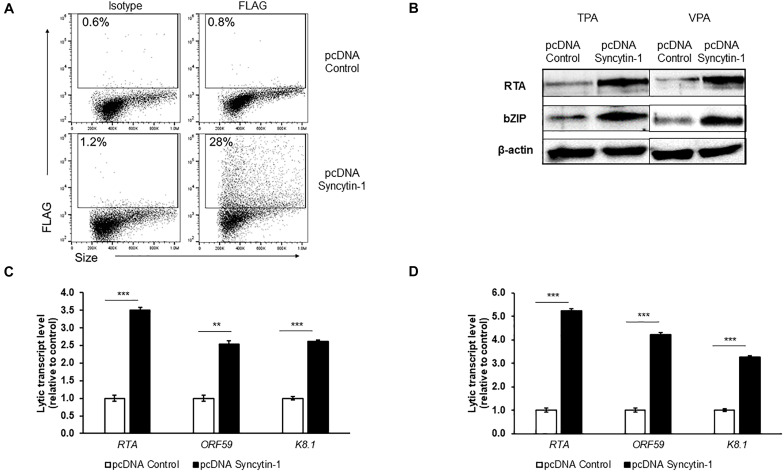
Syncytin-1 enhances KSHV lytic cycle. BCBL-1 cells were nucleofected with empty vector pcDNA or FLAG-tagged pcDNA-Syncytin-1 and harvested at 24 h for flow cytometry to demonstrate Syncytin-1 overexpression via FLAG staining **(A)** or exposed to TPA or VPA to induce the lytic cycle and harvested 24 h **(B)** or 36 h **(C,D)** post induction to assay lytic activation by immunoblotting with antibodies to indicated lytic antigens **(B)** and RT-qPCR of a representative lytic gene of each kinetic class after induction with TPA **(C)** or VPA **(D)**. Data represent averages of three independent experiments; error bars, SEM; **p ≤ 0.01, ***p ≤ 0.001.

To further determine if Syncytin-1 can promote KSHV lytic activation, we overexpressed Syncytin-1 FLAG for 24 h (Figure 6A), followed by TPA or VPA induction of lytic replication. As seen with EBV, KSHV lytic gene analysis showed a significant increase in transcripts in the Syncytin-1 overexpression condition regardless of lytic trigger employed (Figures 6C,D). RTA and bZIP proteins were also enhanced upon Syncytin-1 overexpression (Figure 6B). Once again, while supportive, Syncytin-1 overexpression did not appear to be sufficient to trigger the lytic cascade for KSHV in the absence of a known lytic trigger (Supplementary Figure 4). Taken together with the EBV studies above, the results reveal pro-lytic regulatory effects of Syncytin-1 on both human gammaherpesviruses.

### Syncytin-1 May Contribute to Cellular Proliferation

**FIGURE 7:**
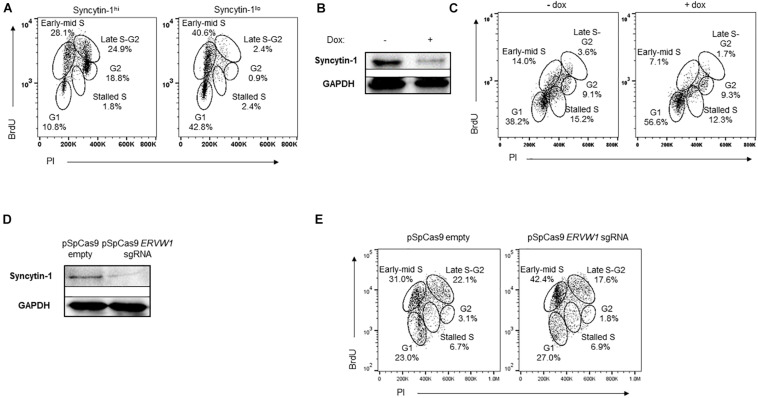
Syncytin-1 supports proliferation of EBV^+^ cells. **(A)** Syncytin-1 positive HH514-16 cells were sub-gated equally into Syncytin-1^*hi*^ and Syncytin-1^*lo*^ sub-populations, which were then further examined for cell cycle analysis via flow cytometry. **(B,C)** A stably integrated doxycycline-inducible Syncytin-1 knockdown HH514-16 cell line was induced with doxycycline or left untreated for 24 h to reduce Syncytin-1 expression followed by western blot to analyze Syncytin-1 levels **(B)** and cell cycle analysis as in **(A)** for each treatment group **(C)**. **(D,E)** HH514-16 cells were nucleofected with pSpCas9 BB-2A-puro or pSpCas9 BB-2A-puro *ERVW-1* guide RNA for 24 h to knockdown Syncytin-1 expression followed by western blot to analyze Syncytin-1 levels **(D)** and cell cycle analysis **(E)**.

Because we observed Syncytin-1 in LCL and BL-derived cell lines, and found that it promotes the lytic phase of human gammaherpesviruses, we further explored its role in B cell biology. Specifically, we explored a potential role for Syncytin-1 in cell cycle progression. Cell cycle analysis was performed via 5-bromo-2′-deoxyuridine (BrdU) uptake and flow cytometry along with Syncytin-1 co-staining. This revealed many more cells in late S and G2 phases among Syncytin-1 high as compared to the Syncytin-1 low cells, which appeared mainly in G1 (Figure 7A). To determine if Syncytin-1 knockdown would impede cell cycle progression, we established a doxycycline-inducible Syncytin-1 knockdown cell line derived from HH514-16 (CLIX-sh*ERVW1*) bearing a plasmid encoding an *ERVW-1*-specific shRNA under an inducible pTET promoter element (pLIX_sh*ERVW1*). Using this cell line, an 18 h treatment with doxycycline substantially reduced Syncytin-1 protein levels as compared to untreated cells (Figure 7B). In cell cycle analysis of CLIX-sh*ERVW1* cells, doxycycline-mediated knockdown of Syncytin-1 indeed resulted in a greater percentage of cells remaining in the G1 phase and unable to progress through the cell cycle. Untreated cells by contrast appeared in all phases of the cell cycle with more cells notably in early to mid S and late S to G2 phases as compared to Syncytin-1 depleted cells (Figure 7C). To further explore the phenotype of Syncytin-1 knockdown on cell cycle progression, we utilized a CRISPR all-in-one plasmid containing a guide RNA to *ERVW-1*. After nucleofecting HH514-16 cells for 24 h and confirming knockdown (Figure 7D), cell cycle analysis was performed. As shown in Figure 7E, cells with a knockdown in Syncytin-1 protein had a larger percentage of cells in the G1 and early S phases as compared to the control.

**FIGURE 8:**
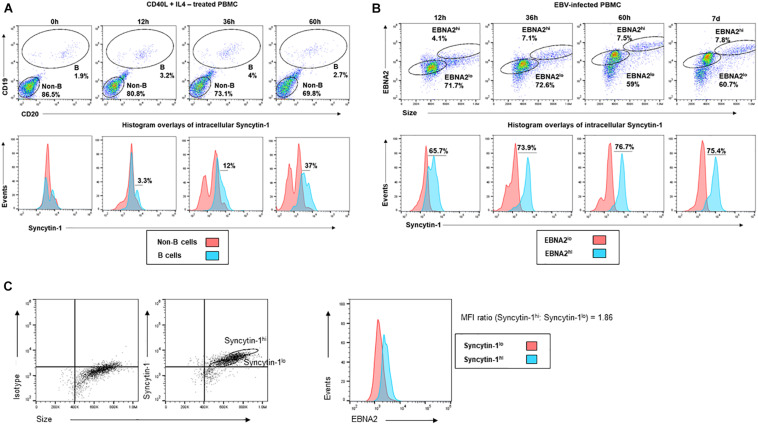
Immune-activated and newly infected primary B cells upregulate Syncytin-1 expression. **(A)** Healthy subject-derived PBMC were exposed to CD40L and IL-4 and harvested at 0, 12, 36, or 60 h for staining with antibodies to CD19 and CD20 (B cell markers) and Syncytin-1 using flow cytometry. Cells were gated for B and non-B cells. Histogram overlays for intracellular Syncytin-1 were plotted for the B cell and non-B cell populations. **(B)** Healthy subject-derived PBMC were infected with EBV and harvested at 0, 36, and 60 h, or 7 days for staining with antibodies to EBNA2 and Syncytin-1 using flow cytometry. Cells were gated for EBNA2^*hi*^ and ^*lo*^ cells populations based on isotype controls. Histogram overlays for intracellular Syncytin-1 were plotted for each population. **(C)** PBMC derived from a kidney transplant patient with LPD were stained for Syncytin-1 and EBNA2 using flow cytometry. Cells were gated into Syncytin-1 high and low populations followed by histogram overlays of EBNA2 for the Syncytin-1 sub-populations. Geometric mean ratio was calculated by normalizing to the Syncytin-1^*lo*^ population.

With the potential for a role for Syncytin-1 in B cell proliferation, we further examined Syncytin-1 expression in PBMC obtained from healthy donors. PBMC were stimulated with CD40L and IL-4 to promote B cell proliferation and collected at 0, 12, 36, and 60 h. At each time point, cells were fixed and stained for flow cytometry using antibodies to the B cell markers CD19 and CD20, as well as Syncytin-1. The resulting staining profiles showed proliferating B cells to contain higher levels of Syncytin-1 than non-B cells (Figure 8A). To investigate a possible association between EBV induced cell proliferation and Syncytin-1, a similar analysis was performed on EBV infected PBMC, known to result in B cell proliferation, transformation, and establishment of EBV latency. PBMC were infected with EBV and harvested at 12, 36, and 60 h, and 7 days post infection, co-stained for Syncytin-1 and EBNA2 (EBV nuclear antigen 2, essential for driving B cell proliferation and latency), and analyzed by flow cytometry. This revealed Syncytin-1 levels to be consistently higher in EBNA2hi cells compared to EBNA2lo cells (Figure 8B), indicating a role for Syncytin-1 in facilitating the establishment and outgrowth of EBV-transformed lymphoblastoid cells in addition to its role in promoting lytic virus production.

In assessing if Syncytin-1 was indeed expressed in B cells in blood and contributed to EBV-latency, we examined peripheral blood mononuclear cells (PBMC) obtained from a transplant recipient with EBV-lymphoproliferative disease. We stained cells for Syncytin-1 and EBNA2 to determine if there was an association between Syncytin-1 and EBV positive and/or proliferating cells (Figure 8C). This revealed cells high in Syncytin-1 expression also expressed higher levels of EBNA2, with a 1.86 fold greater difference in the geometric mean of Syncytin-1^*hi*^ cells compared to Syncytin-1^*lo*^ cells (Figure 8C). Overall, these data provide evidence for a potential role of Syncytin-1 in B cell proliferation.

## Discussion

Viruses causing persistent infections, such as retro- and herpesviruses, are well-adapted manipulators of host cellular processes. Gammaherpesviruses, EBV, and KSHV in particular, exploit numerous host pathways and factors to regulate their latent and lytic states (Kenney and Mertz, 2014; Murata, 2014; Li and Bhaduri-McIntosh, 2016; Li et al., 2017, 2018; Broussard and Damania, 2020; Burton et al., 2020; Campbell et al., 2020; Frey et al., 2020; Bhaduri-McIntosh and McIntosh, 2021). For instance, both viruses usurp host epigenetic machinery to heterochromatinize and silence their own genomes during latency (Scott, 2017; Pei et al., 2020; Bhaduri-McIntosh and McIntosh, 2021), and both exploit host signal transduction pathways and host transcription factors to activate from latency (Kenney and Mertz, 2014; Aneja and Yuan, 2017; Broussard and Damania, 2020).

In this study, we asked if HERVs and persistent gammaherpesvirus infections influenced each other’s expression during lytic activation of the gammaherpesvirus from latency. This initially revealed an increase in certain HERV/LTR elements including *ERVW-1* in both nascent and steady state mRNA in BL cells harboring lytic replicating EBV as compared to latently infected BL cells. This was notable given the well-known global inhibition of host gene expression during lytic replication in a mechanism of accelerated host mRNA turnover, common to herpesviruses and termed “host shutoff” (Rowe et al., 2007; Covarrubias et al., 2009; Clyde and Glaunsinger, 2011). While our initial focus on Syncytin-1 arose from our observation of activated *ERVW-1* transcription during the lytic phase of EBV (Supplementary Table 1), its presence as a protein in B lymphoma cells and replicating B cell blasts suggests an underlying role in B-cell biology. Prior comparative studies between healthy individuals and MS patients similarly report the expression of Syncytin-1 or a close homolog in astrocytes and peripheral blood monocytes, NK, and B cells, but not T cells using RT-qPCR and flow cytometry methods (Mameli et al., 2012, 2013). Importantly, Syncytin-1 and its homolog MSRV*env* can be distinguished by differential PCR and protein studies, allowing for high specificity (Mameli et al., 2009; Charvet et al., 2021). Similarly, elevated HERV-W transcripts were previously observed upon primary lytic infection of herpes simplex virus-1 (Nellaker et al., 2006), and elevated *HERV-K18 env* gene transcription was likewise noted upon primary infection of BJAB lymphoma cells with EBV (Sutkowski et al., 2001). Similarly, Syncytin-1 was found to be transactivated by HSV-1 infection in neuronal and brain endothelial cells (Ruprecht et al., 2006). Our studies on transcripts, while demonstrating upregulation of the *ERVW-1* locus, did not show abundant active transcription despite a detectable level of Syncytin-1 protein by flow cytometry and western blot. This suggests a low basal level of transcription for *ERVW-1* and relative stability of the Syncytin-1 protein. In this regard, we found HERV elements *ERV3-1*, encoding a Syncytin-1 homolog, and *ERVK13-1*, encoding a lincRNA, to be transcribed at higher rates in BL cells with or without induction of the EBV lytic cycle (Supplementary Table 1).

*ERVW-1* siRNA knockdown resulted in a substantial reduction in Syncytin-1 detection by western blot and a significant reduction in expression of EBV and KSHV lytic genes of each kinetic class (Figures 2, 5). In this regard, human gammaherpesviruses appear to share a dependence on Syncytin-1 for activation of their lytic programs. This is in contrast to progression through the lytic phase marked by viral DNA replication, particle assembly and virus egress. As these later events are all downstream of expression of the immediate early latent-to-lytic switch proteins ZEBRA, for EBV, and RTA, for KSHV, their inhibition may be more a consequence of less activation of the lytic phase rather than direct involvement of Syncytin-1 with those later processes. Additionally, cell-to-cell fusions are not readily observed in BL cultures or B cell blasts and Syncytin-1 expression appears far less than that observed in placental trophoblasts, pointing to a non-fusogenic role for Syncytin-1 in these cells. CRISPR-Cas9 targeted knockout of the *ERVW-1* locus was successful in HH514-16 BL cells, but a stable knockout line was unable to proliferate and establish, likely due to a required role for Syncytin-1 in B cell proliferation. Instead, we turned to an inducible shRNA directed knockdown of *ERVW-1* that showed a substantial reduction in cells exiting G1 and or progressing through the cell cycle (Figure 7). This is similar to recent observations in placental trophoblast-derived cell cultures where knockdown of Syncytin-1 also results in G1/S transition arrest (Huang et al., 2013, 2014). CRISPR-mediated depletion of Syncytin-1 also showed a defect in BL cell proliferation (Figure 7E), though the defect was less pronounced at the G1/S transition with more cells instead stalled in the S phase. In this regard, varying efficiencies of Syncytin-1 depletion may differentially influence the cells ability to enter the S phase versus its ability to traverse the S phase. Further supporting a role for Syncytin-1 in B cell proliferation is the observation that BL cells with naturally low Syncytin-1 levels also appeared far more abundant in the G1 and early S phases as compared to Syncytin-1 high cells (Figure 7A). As Syncytin-1, a cytoplasmic and plasma membrane protein, does not appear to localize to the nucleus, we hypothesize that its impact on B cell proliferation suggests a role in stimulating signal transduction pathways, perhaps via mitogen-activated protein kinases (MAPKs), protein kinase C (PKC), or phosphoinositide 3-kinase/Akt pathway (PI3K/Akt). While adverse cellular effects of Syncytin-1 depletion likely contribute to the reduction of lytic activation of EBV or KSHV in depletion studies, augmentation of lytic activation upon overexpression of Syncytin-1 supports a role for Syncytin-1 in establishing a pyrolytic environment.

Various external or internal triggers of human gammaherpesvirus lytic replication, such as B cell receptor engagement or exposure to phorbol esters, stimulate signal transduction via the MAPK, PKC, or PI3K/Akt signaling pathways. These converge to activate host transcription factors that then translocate to the nucleus and activate expression of genes involved in cell differentiation, proliferation, and survival, as well as latent-to-lytic switch genes ZEBRA and/or RTA (Kenney and Mertz, 2014; Broussard and Damania, 2020). In this regard, Syncytin-1 may be functioning like other exogenous or endogenous retroviral envelope proteins, such as that of the oncogenic Jaagsiekte sheep retrovirus (JSRV) linked to pulmonary adenocarcinoma in sheep (Palmarini et al., 1999; Wootton et al., 2005; Caporale et al., 2006). The cytoplasmic tail of the JSRV envelope protein is known to interact with host factors leading to constitutive stimulation of MAPK and PI3K/Akt signaling pathways that in turn result in activation of pro-survival and pro-proliferative pathways leading to cellular transformation and cancer (Hull and Fan, 2006; Liu and Miller, 2007). Similarly, HERV envelope proteins like Syncytin-1 and others may stimulate pro-survival and pro-proliferative pathways in cells beyond the canonical roles ascribed to them in mediating cell-to-cell fusions. In this regard, human gammaherpesviruses appear to exploit such signaling to not only persist in latency via survival and proliferation of their host B cell, but also redirect such signaling to promote activation of the lytic replicative phase when cued to escape. In the specific examples of EBV and KSHV, Syncytin-1 appears to contribute as a pro-lytic factor. Overexpression of Syncytin-1, while enhancing the number of cells able to respond to lytic trigger, however, did not spark lytic replication of EBV or KSHV in the absence of an exogenous lytic trigger (Supplementary Figures 3, 4). Thus, Syncytin-1 appears to be necessary to the lytic activation of human gammaherpesviruses while not sufficient to trigger the lytic phase.

Collectively therefore, these findings suggest that human gammaherpesviruses and B cells have developed a dependency on Syncytin-1. This dependency not only facilitates the ability of EBV and KSHV to activate lytic replication from latency, but also promotes their persistence in the latent state by contributing to B cell progression through the cell cycle.

## Data Availability Statement

The datasets presented in this study can be found in online repositories. The names of the repository/repositories and accession number(s) can be found below: https://www.ncbi.nlm.nih.gov/, GSE141220.

## Ethics Statement

The studies involving human participants were reviewed and approved by the Institutional Review Board of the University of Florida. Written informed consent to participate in this study was provided by the participants’ legal guardian/next of kin.

## Author Contributions

TF and MM designed the study and wrote the manuscript. TF, IA, and EB acquired the data. TF, IA, SB-M, and MM interpreted the data. All authors contributed to the article and approved the submitted version.

## Conflict of Interest

The authors declare that the research was conducted in the absence of any commercial or financial relationships that could be construed as a potential conflict of interest.

## Publisher’s Note

All claims expressed in this article are solely those of the authors and do not necessarily represent those of their affiliated organizations, or those of the publisher, the editors and the reviewers. Any product that may be evaluated in this article, or claim that may be made by its manufacturer, is not guaranteed or endorsed by the publisher.
