# Supplementary material for: An Ancestral Retrovirus Envelope Protein Regulates Persistent Gammaherpesvirus Lifecycles
Source: Front Microbiol. 2021 Aug 9;12:708404. doi: 10.3389/fmicb.2021.708404 (PMC8381357; doi:10.3389/fmicb.2021.708404)
Supplement: Supplementary file 1 [file Table_1.DOCX]

| **Induction**  **Time NaB** | **Repeat/Locus** | **Size (nt)** | **No. of Loci** | **Class/Family** | **FC** | **Log2FC** | **Uninduced Mean Normalized Reads** | **Induced Mean Normalized Reads** |
| --- | --- | --- | --- | --- | --- | --- | --- | --- |
| **3 hr** | HY3 | 93 | 517 | scRNA | 2.51 | 1.33 | 3.00 | 7.53 |
|  | LTR12C | 1579 | 2623 | LTR/ERV1 | 2.23 | 1.16 | 29.25 | 65.15 |
|  | LSAU | 498 | 148 | Satellite | 2.19 | 1.13 | 15.50 | 34.00 |
|  | ERV3-1 | 15845 | 1 | Protein coding | 1.26 | 0.33 | 2725.58 | 3437.60 |
|  | ERVFRD-3 | 1617 | 1 | Pseudogene | 0.50 | -1.00 | 44.16 | 22.02 |
|  | ERVK13-1 | 13093 | 1 | Transcript | 1.08 | 0.11 | 1472.22 | 1592.57 |
| **24 hr** | HSATI | 568 | 57 | Satellite | 1121.0 | 10.13 | 0.00 | 112.10 |
|  | SAR | 84 | 438 | Satellite | 77.12 | 6.27 | 6.25 | 481.99 |
|  | SATR1 | 423 | 5122 | Satellite | 2.93 | 1.55 | 8.00 | 23.44 |
|  | CER | 96 | 8547 | Satellite | 2.85 | 1.51 | 8.00 | 22.77 |
|  | BSR/Beta | 152 | 3137 | Satellite | 2.61 | 1.38 | 540.75 | 1412.14 |
|  | D20S16 | 98 | 1980 | Satellite | 2.51 | 1.33 | 15.25 | 38.25 |
|  | LSAU | 498 | 148 | Satellite | 2.17 | 1.12 | 45.50 | 98.83 |
|  | U5 | 116 | 83 | snRNA | 201.20 | 7.65 | 0.00 | 20.12 |
|  | ALR/Alpha | 171 | 43645 | Satellite/centr | 35.79 | 5.16 | 17.50 | 626.37 |
|  | SST1 | 1563 | 784 | Satellite/centr | 3.78 | 1.92 | 2.75 | 10.39 |
|  | LTR12C | 1579 | 2623 | LTR/ERV1 | 7.12 | 2.83 | 21.75 | 154.77 |
|  | HERV9NC-int | 6118 | 314 | LTR/ERV1 | 6.27 | 2.65 | 8.00 | 50.19 |
|  | HERV9-int | 8436 | 427 | LTR/ERV1 | 4.60 | 2.20 | 6.25 | 28.74 |
|  | MER41E | 595 | 378 | LTR/ERV1 | 4.38 | 2.13 | 4.75 | 20.78 |
|  | LTR39 | 783 | 1267 | LTR/ERV1 | 2.34 | 1.23 | 4.25 | 9.95 |
|  | LTR12D | 1254 | 2013 | LTR/ERV1 | 3.50 | 1.81 | 15.75 | 55.05 |
|  | LOR1-int | 8120 | 1177 | LTR/ERV1 | 3.32 | 1.73 | 6.00 | 19.90 |
|  | MER52D | 2123 | 1183 | LTR/ERV1 | 2.95 | 1.56 | 3.75 | 11.05 |
|  | LTR1A2 | 837 | 971 | LTR/ERV1 | 2.42 | 1.28 | 4.75 | 11.50 |
|  | MER34B | 565 | 503 | LTR/ERV1 | 2.18 | 1.12 | 3.25 | 7.08 |
|  | HERVE-int | 7813 | 238 | LTR/ERV1 | 2.15 | 1.11 | 5.75 | 12.38 |
|  | MER34A1 | 587 | 822 | LTR/ERV1 | 2.13 | 1.09 | 4.25 | 9.06 |
|  | LTR8A | 727 | 1376 | LTR/ERV1 | 2.10 | 1.07 | 6.00 | 12.60 |
|  | HERV9N-int | 5374 | 443 | LTR/ERV1 | 2.03 | 1.02 | 17.50 | 35.60 |
|  | HERVIP10FH-int | 5102 | 464 | LTR/ERV1 | 2.03 | 1.02 | 10.25 | 20.78 |
|  | LTR9 | 612 | 801 | LTR/ERV1 | 2.02 | 1.02 | 3.50 | 7.08 |
|  | MLT1F | 548 | 4429 | LTR/ERVL-MaLR | 4.23 | 2.08 | 2.25 | 9.51 |
|  | MST-int | 1651 | 3857 | LTR/ERVL-MaLR | 2.10 | 1.07 | 23.75 | 49.97 |
|  | HERVK11D-int | 7752 | 54 | LTR/ERVK | 3.12 | 1.64 | 290.00 | 905.83 |
|  | HERVK14C-int | 7434 | 197 | LTR/ERVK | 2.59 | 1.37 | 101.00 | 261.34 |
|  | HERVK-int | 7536 | 254 | LTR/ERVK | 2.31 | 1.21 | 133.50 | 308.21 |
|  | HERVK14-int | 6096 | 503 | LTR/ERVK | 2.09 | 1.06 | 84.25 | 175.99 |
|  | HERVK13-int | 8116 | 72 | LTR/ERVK | 2.04 | 1.03 | 144.75 | 295.16 |
|  | LTR13A | 966 | 195 | LTR/ERVK | 2.01 | 1.01 | 4.50 | 9.06 |
|  | Charlie5 | 2624 | 2622 | DNA/hAT-Charlie | 2.97 | 1.57 | 3.50 | 10.39 |
|  | Tigger5 | 2406 | 172 | Tigger | 2.38 | 1.25 | 3.25 | 7.74 |
|  | L1M3c | 1625 | 1166 | LINE/L1 | 2.51 | 1.33 | 3.00 | 7.52 |
|  | ERV3-1 | 15845 | 1 | Protein coding | 0.78 | -0.37 | 2652.41 | 2057.07 |
|  | ERVFRD-1 | 9244 | 1 | Protein coding | 19.32 | 4.27 | 0.50 | 9.66 |
|  | ERVFRD-3 | 1617 | 1 | Pseudogene | 0.19 | -2.37 | 46.65 | 9.01 |
|  | ERVH-1 | 10318 | 1 | LincRNA | 165.81 | 7.37 | 0.00 | 16.58 |
|  | ERVH48-1 | 8844 | 1 | LincRNA | 10.30 | 3.36 | 0.50 | 5.15 |
|  | ERVK13-1 | 13093 | 1 | Transcript | 0.54 | -0.88 | 1118.27 | 606.41 |
|  | ERVMER34-1 | 9089 | 1 | Protein coding | 12.90 | 3.69 | 1.08 | 13.95 |
|  | ERVV-2 | 6339 | 1 | Protein coding | 73.71 | 6.20 | 0.00 | 7.37 |
|  | ERVW-1 | 9536 | 1 | Protein coding | 199.52 | 7.64 | 0.00 | 19.95 |
| **48 hr** | HSATI | 568 | 57 | Satellite | 91.61 | 6.52 | 0.50 | 45.81 |
|  | SAR | 84 | 438 | Satellite | 5.25 | 2.39 | 33.50 | 175.96 |
|  | ALR/Alpha | 171 | 43645 | Satellite/centr | 23.78 | 4.57 | 11.50 | 273.48 |
|  | SST1 | 1563 | 784 | Satellite/centr | 7.71 | 2.95 | 1.25 | 9.63 |
|  | SSU-rRNA_Hsa | 1869 | 67 | rRNA | 4.28 | 2.10 | 1662.00 | 7110.07 |
|  | LSU-rRNA_Hsa | 5035 | 235 | rRNA | 2.59 | 1.37 | 2023.25 | 5238.11 |
|  | U5 | 116 | 83 | snRNA | 3.21 | 1.68 | 4.00 | 12.85 |
|  | LOR1-int | 8120 | 1177 | LTR/ERV1 | 2.42 | 1.27 | 4.75 | 11.49 |
|  | HERV9-int | 8436 | 427 | LTR/ERV1 | 2.38 | 1.25 | 7.25 | 17.24 |
|  | HERV9NC-int | 6118 | 341 | LTR/ERV1 | 2.14 | 1.10 | 10.25 | 21.97 |
|  | ERV3-1 | 15845 | 1 | Protein coding | 0.33 | -1.60 | 3103.53 | 1026.47 |
|  | ERVFRD-1 | 9244 | 1 | Protein coding | 12.86 | 3.68 | 0.49 | 6.28 |
|  | ERVFRD-3 | 1617 | 1 | Pseudogene | 0.42 | -1.25 | 20.58 | 8.65 |
|  | ERVH-1 | 10318 | 1 | LincRNA | 213.92 | 7.74 | 0.00 | 21.39 |
|  | ERVK13-1 | 13093 | 1 | Transcript | 0.84 | -0.25 | 866.06 | 726.16 |
|  | ERVMER34-1 | 9089 | 1 | Protein coding | 18.96 | 4.24 | 0.85 | 16.05 |
|  | ERVV-2 | 6339 | 1 | Protein coding | 165.54 | 7.37 | 0.00 | 16.55 |
|  | ERVW-1 | 9536 | 1 | Protein coding | 151.15 | 7.24 | 0.00 | 15.11 |

**Supplemental Table 1. Fold change in nascent transcripts of repeat regions during lytic induction of EBV.** Nascent transcription was previously determined using Bru-Seq at 3, 24, and 48 hr with (Induced) and without (Uninduced) exposure of EBV+ HH514-16 cells to the lytic inducing agent, sodium butyrate (NaB), as previously described (Frey, et al., 2020), and unmapped reads to the human genome GRCh38 were aligned to the RepeatMasker database to determine nascent transcription of repeat elements. Differentially expressed repeat elements with greater than 2 fold induction and a mean read count of 5 or greater are displayed (Shaded). Additionally, individual genetic loci encoding HERV elements annotated in the human genome with high read counts or 2 fold or greater induction are also shown (Unshaded). Fold change (FC) was calculated as the ratio of induced to uninduced reads. Data are normalized to total reads in uninduced control samples and represent the means of two independent experiments. Primary sequence and gene expression data are deposited at the Geodatabase, accession number GSE141220. Identities of each element are available at <http://www.dfam.org/browse>. Number of loci represent the trusted threshold count of non-redundant hits within the human genome (GRCh38) annotated in the Dfam database.
