# Supplementary material for: An Ancestral Retrovirus Envelope Protein Regulates Persistent Gammaherpesvirus Lifecycles
Source: Front Microbiol. 2021 Aug 9;12:708404. doi: 10.3389/fmicb.2021.708404 (PMC8381357; doi:10.3389/fmicb.2021.708404)
Supplement: Supplementary file 2 [file Image_1.pdf]

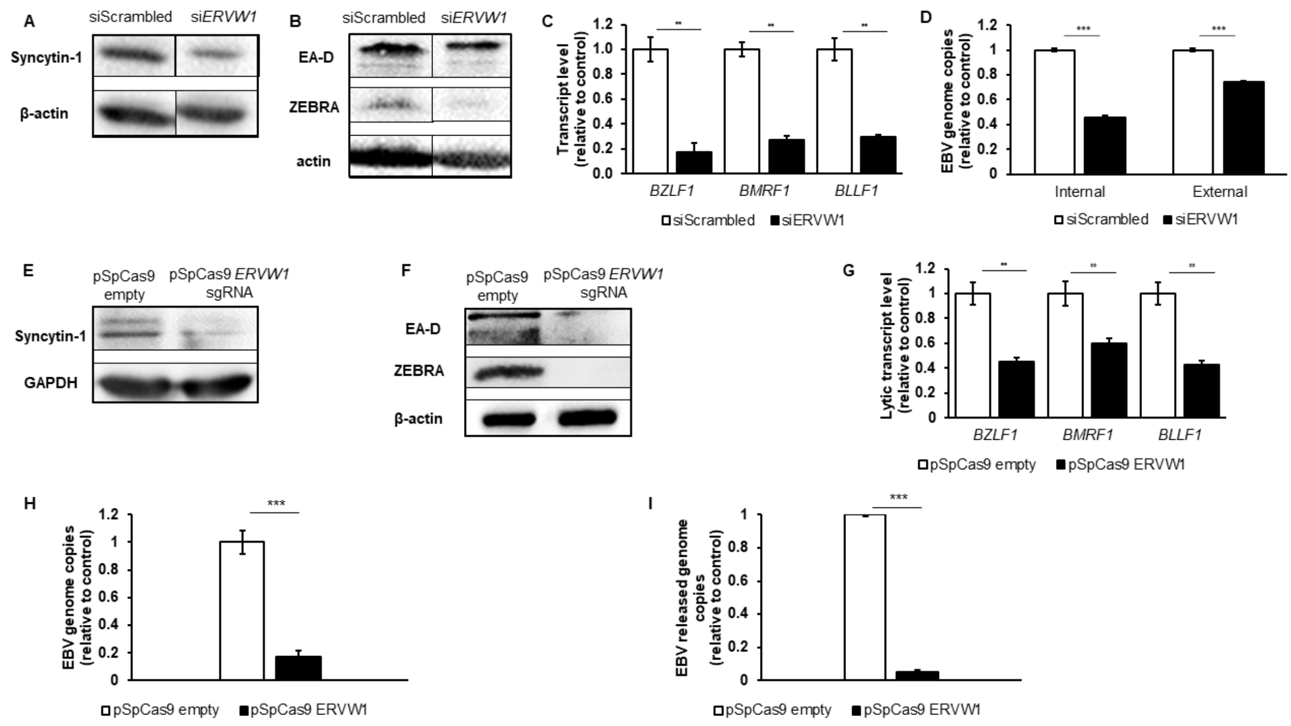

Supplemental Figure 1. Syncytin-1 knockdown impairs lytic activation in HH514-16 BL cells. HH514-16 cells were nucleofected with scrambled control or *ERVW1* specific siRNAs for 18 hours and harvested for western blot analysis for Syncytin-1 **(A)** or treated with NaB to induce lytic activation and harvested at 24 **(B)**, 36 **(C, D)** or 72 **(D)** hr to analyze lytic readouts in each siRNA treatment group. Lytic proteins EA-D and ZEBRA were probed using western blot in **(B)**. Lytic transcript levels of representative genes of each EBV kinetic class were assayed using RT-qPCR in **(C)**. **(D)** EBV *BamW* qPCR was performed on DNA isolated from cell pellets (left, internal) or DNase-treated, filtered cell culture medium (right, external). **(E-I)** HH514-16 cells were nucleofected with pSpCas9 BB-2A-puro or pSpCas9 BB-2A-puro *ERVW1* guide RNA for 24 hr followed by harvest for western blot analysis **(E)** or treatment NaB to induce lytic activation. Cells were harvested at 24 **(F)**, 36 **(G, H)**, or 72 **(I)** hr to analyze lytic readouts as in B, C, and D, respectively. Data represent averages of three independent experiments; error bars, SEM; $^{**}p \leq 0.01$, $^{***}p \leq 0.001$.

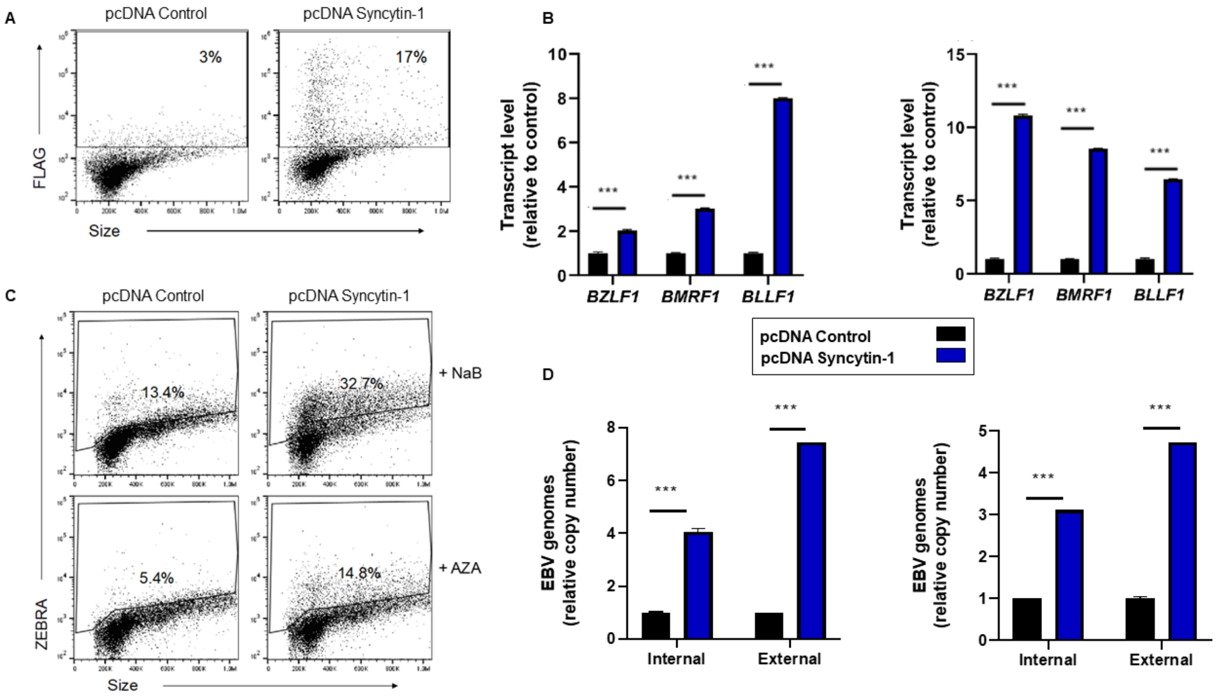

Supplemental Figure 2. Syncytin-1 enhances lytic activation in HH514-16 BL cells. HH514-16 cells were nucleofected with empty vector pcDNA or FLAG-tagged pcDNA-Syncytin-1 and harvested 24 hr later for flow cytometry to demonstrate Syncytin-1 overexpression via FLAG staining **(A)** or exposed to NaB or AZA to induce the lytic cycle and harvested after another 24 **(C)**, 36 **(B)**, or 72 **(D)** hr to assay lytic activation by RT-qPCR of a representative lytic gene of each kinetic class **(B)**, flow cytometry with antibodies against ZEBRA lytic antigens **(C)**, or cell-associated viral load (internal) and DNase-resistant released virions (external) **(D)**. Data represent averages of three independent experiments; error bars, SEM; ***$p \leq 0.001$.

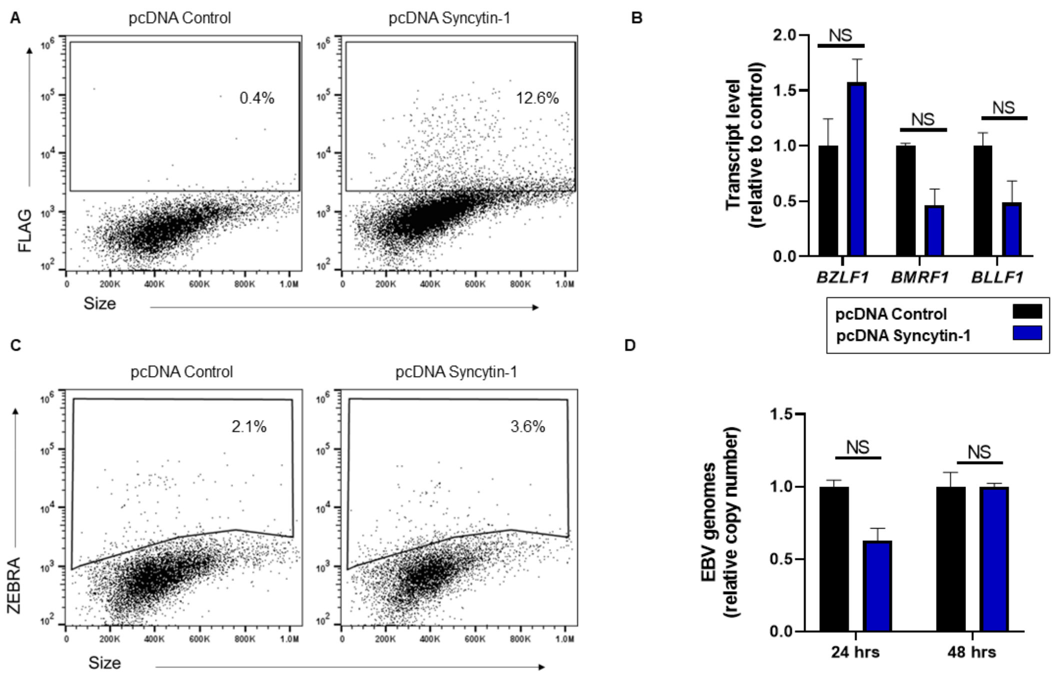

Supplemental Figure 3. Syncytin-1 overexpression is not sufficient to activate the EBV lytic cycle. HH514-16 cells were nucleofected with empty vector pcDNA or FLAG-tagged pcDNA-Syncytin-1 and harvested 24 hr later for flow cytometry to demonstrate Syncytin-1 overexpression via FLAG staining **(A)** or left unexposed to lytic triggers to determine spontaneous lytic activation and harvested after another 24 **(C)** or 36 **(B, D)** hr to assay lytic activation by RT-qPCR of a representative lytic gene of each kinetic class **(B)**, flow cytometry with antibodies against ZEBRA lytic antigens **(C)**, or cell-associated viral load **(D)**. Data represent averages of three independent experiments; error bars, SEM; NS = not significant.

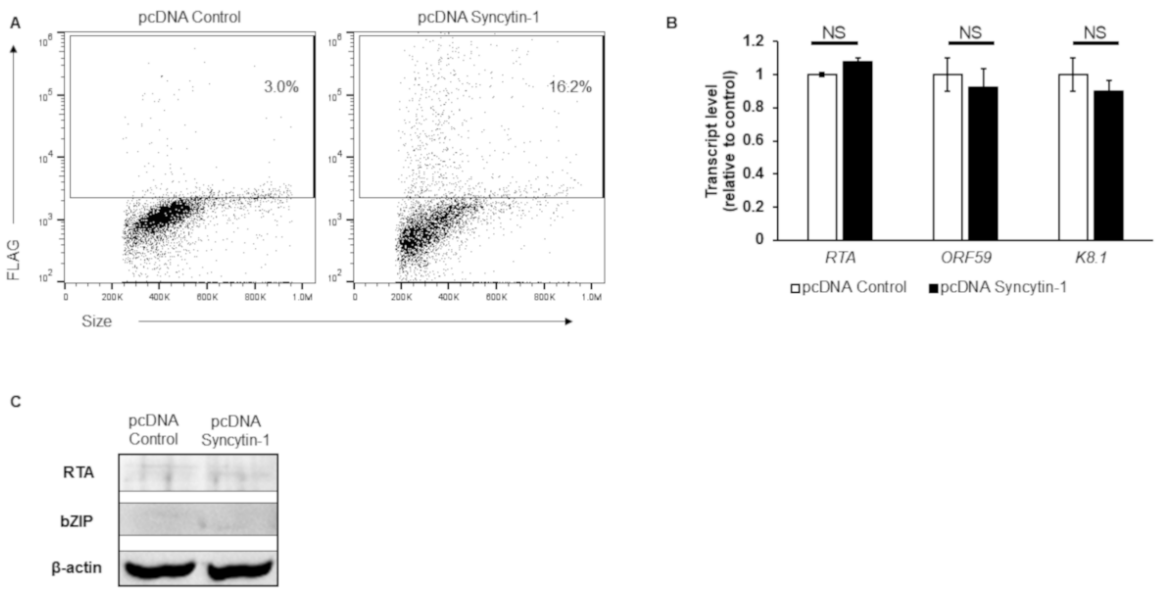

Supplemental Figure 4. Syncytin-1 overexpression is not sufficient to activate the KSHV lytic cycle. BCBL-1 cells were nucleofected with empty vector pcDNA or FLAG-tagged pcDNA-Syncytin-1 and harvested 24 hr later for flow cytometry to demonstrate Syncytin-1 overexpression via FLAG staining **(A)** or left unexposed to lytic triggers to determine spontaneous lytic activation and harvested after another 24 **(C)** or 36 **(B)** hr to assay lytic activation by RT-qPCR of a representative lytic gene of each kinetic class **(B)** or western blot with antibodies against KSHV lytic antigens **(C)**. Data represent averages of three independent experiments; error bars, SEM; NS = not significant.
